# *QuickStats:* Percentage* of Persons Who Used Telemedicine During the Past 12 Months,^†^ by Age Group — National Health Interview Survey, United States, 2021^§^

**DOI:** 10.15585/mmwr.mm7205a2

**Published:** 2023-02-03

**Figure:**
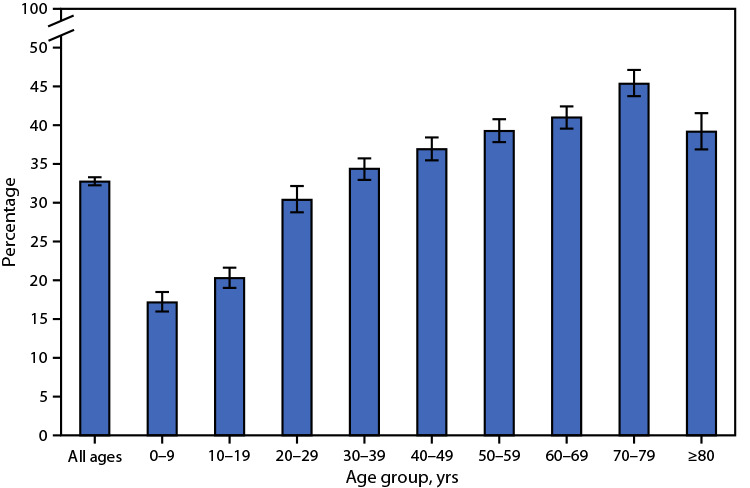
In 2021, approximately one third (32.8%) of persons of all ages had a telemedicine appointment with a doctor, nurse, or other health professional during the past 12 months. The percentage with a telemedicine appointment increased with age, from 17.2% among children aged <10 years to 45.5% among adults aged 70–79 years, and then decreased to 39.3% among adults aged ≥80 years. Telemedicine use among adults aged ≥80 years was similar to that among adults aged 40–49, 50–59, and 60–69 years.

**Source:** National Center for Health Statistics, National Health Interview Survey, 2021. https://www.cdc.gov/nchs/nhis.htm

